# Network for Early Onset Cystic Kidney Diseases—A Comprehensive Multidisciplinary Approach to Hereditary Cystic Kidney Diseases in Childhood

**DOI:** 10.3389/fped.2018.00024

**Published:** 2018-02-13

**Authors:** Jens Christian König, Andrea Titieni, Martin Konrad, The NEOCYST Consortium, C. Bergmann

**Affiliations:** ^1^Department of General Pediatrics, University Children’s Hospital Münster, Münster, Germany

**Keywords:** hereditary cystic kidney diseases, ciliopathy, nephronophthisis, autosomal recessive polycystic kidney disease, Bardet–Biedl syndrome, hepatocyte nuclear factor-1beta nephropathy

## Abstract

Hereditary cystic kidney diseases comprise a complex group of genetic disorders representing one of the most common causes of end-stage renal failure in childhood. The main representatives are autosomal recessive polycystic kidney disease, nephronophthisis, Bardet–Biedl syndrome, and hepatocyte nuclear factor-1beta nephropathy. Within the last years, genetic efforts have brought tremendous progress for the molecular understanding of hereditary cystic kidney diseases identifying more than 70 genes. Yet, genetic heterogeneity, phenotypic variability, a lack of reliable genotype–phenotype correlations and the absence of disease-specific biomarkers remain major challenges for physicians treating children with cystic kidney diseases. To tackle these challenges comprehensive scientific approaches are urgently needed that match the ongoing “revolution” in genetics and molecular biology with an improved efficacy of clinical data collection. Network for early onset cystic kidney diseases (NEOCYST) is a multidisciplinary, multicenter collaborative combining a detailed collection of clinical data with translational scientific approaches addressing the genetic, molecular, and functional background of hereditary cystic kidney diseases. Consisting of seven work packages, including an international registry as well as a biobank, NEOCYST is not only dedicated to current scientific questions, but also provides a platform for longitudinal clinical surveillance and provides precious sources for high-quality research projects and future clinical trials. Funded by the German Federal Government, the NEOCYST collaborative started in February 2016. Here, we would like to introduce the rationale, design, and objectives of the network followed by a short overview on the current state of progress.

## Background

Hereditary cystic kidney diseases comprise a group of slowly progressive, chronically debilitating disorders with a high level of complexity. Despite rare individually, as a group they represent one of the most common causes of end-stage renal failure in childhood and, therefore, have an enormous socioeconomic impact. The main representatives of this group of diseases are autosomal recessive polycystic kidney disease (ARPKD), nephronophthisis and nephronophthisis-related ciliopathies (NPH/NPH-RC), Bardet–Biedl syndrome (BBS), and hepatocyte nuclear factor-1beta (HNF1B) nephropathy (1). The incidence ranges from 1:5,000 to 1:100,000 and the estimated overall prevalence is about 300–450 children in Germany (2–7).

Most early onset cystic kidney diseases are inherited as an autosomal recessive trait. One important exception is the HNF1B nephropathy, which follows autosomal dominant inheritance (6). Within the past 20 years scientific efforts have brought tremendous progress for the molecular understanding of hereditary cystic kidney diseases. To date, mutations in more than 70 individual genes have been identified (Table 1) (8). Yet, genetic heterogeneity is a major problem since, there is a significant amount of genetic as well as phenotypic overlap and reliable data on genotype–phenotype correlations are still lacking (9). Moreover, the phenotypic spectrum covered by cystic kidney diseases is highly complex and extremely variable. Although all disease entities are characterized by the development of renal cysts as a common characteristic, clinical courses as well as the onset of chronic renal failure differ significantly. Furthermore, most phenotypes are not limited to the kidneys, but comprise extrarenal organ manifestations (10, 11).

- ARPKD: the clinical presentation of ARPKD is determined by grossly enlarged polycystic kidneys usually diagnosed before birth. Depending on prenatal kidney function, an early oligohydramnion may lead to pulmonary hypoplasia resulting in a perinatal mortality of about 20% in ARPKD. Children surviving the first months of life usually present a preserved renal function for several years. There is obligate hepatic involvement and the clinical presentation of some patients is dominated by a hepatic phenotype rather than by chronic kidney disease (2, 3, 12–14).
- NPH: reduced urinary concentrating capacity, polyuria/polydipsia, and a slowly progressive loss of renal function are the clinical hallmarks of NPH. Urine analysis is typically unremarkable (4). In contrast to ARPKD, kidney volumes are small or normal, and renal cysts have no obligatory feature (15). NPH can be limited to the kidneys, but extrarenal manifestations are found in 20–40% of patients depending on the underlying genetic defect (10). Moreover, many complex syndromes encompass the renal phenotype with NPH, including Senior–Løken syndrome, Joubert-like syndromes, and other NPH-related ciliopathies (16, 17).
- BBS: Bardet–Biedl syndrome is a complex genetic disorder clinically characterized by progressive visual impairment, obesity, developmental delay, postaxial hexadactyly, hypogonadism, and slowly progressive CKD. The clinical presentation is highly variable and can be accompanied by further organ involvement (5). The renal phenotype usually resembles NPH, including polyuria and small- to normal-sized kidneys showing cystic lesions at the corticomedullary junction. However, renal involvement is not mandatory and reported in only 31–42% of BBS (18).
- HNF1B nephropathy: HNF1B is a transcription factor playing a central role in the tissue-specific regulation of gene expression in various organs, such as kidney, liver, biliary duct, pancreas, and genital organs. Initially, mutations in the *HNF1B* gene were described in association with maturity onset diabetes of the young (MODY type 5). Subsequently, it was shown that in patients with congenital cystic renal dysplasia *HNF1B* mutations could frequently be identified (renal cysts and diabetes syndrome, RCAD). In contrast to the NPH spectrum, renal cysts may already be detected prenatally, but renal ultrasound presentation is variable including unilateral and bilateral renal dysplasia with different numbers and sizes of cysts. Depending on the extent of renal dysplasia some patients develop renal failure in early childhood while others preserve normal renal function for their whole life (6). Beyond the renal involvement, the clinical spectrum comprises elevated liver enzymes, hyperuricemia, genital tract malformations, and electrolyte disturbances, such as hypomagnesemia and hypokalemia (11).

**Table 1:** Update on genes identified to cause early onset cystic kidney diseases (2, 10, 19–25).

Nephronophthisis (NPH)(~63% rate of genetic proof)	**Genes accounting for more than 1% of cases**	**Genes accounting for less than 1% of cases**
	NPHP1 (NPHP1/SLS1/JBTS4) (20–39%) INVS (NPHP2) NPHP3 (NPHP3/MKS7) NPHP4 (NPHP4/SLS4) IQCB1 (NPHP5) CEP290 [NPHP6/SLS6/JBTS5/MKS4/Bardet–Biedl syndrome (BBS)14] TMEM67 (NPHP11/JBTS6/MKS3/COACH)	GLIS2 (NPHP7) RPGRIP1L (NPHP8/JBTS7/MKS5/COACH) NEK8 (NPHP9) SDCCAG8 (NPHP10/SLS7/BBS16) TTC21B (NPHP12/JBTS11/SRTD4) WDR19 (NPHP13/SLS8/SRTD5) ZNF423 (NPHP14/JBTS19) CEP164 (NPHP15) ANKS6 (NPHP16) IFT172 (NPHP17/SRTD10) CEP83 (NPHP18) DCDC2 (NPHP19) MAPKBP1 (NPHP20)
Joubert Syndrome(62–94% rate of genetic proof)	**Genes accounting for more than 1% of cases**	**Genes accounting for less than 1% of cases**
	INPP5E (JBTS1) TMEM216 (JBTS2/MKS2) AHI1 (JBTS3) NPHP1 (JBTS4/NPHP1/SLS1) CEP290 (JBTS5/NPHP6/SLS6/MKS4/BBS14) TMEM67 (JBTS6/NPHP11/MKS3/COACH) RPGRIP1L (JBTS7/NPHP8/MKS5/COACH) CC2D2A (JBTS9/MKS6) C5orf42 (JBTS17/OFD6) CSPP1 (JBTS21) KIAA0586 (JBTS23/SRTD14) TCTN2 (JBTS24/MKS8) MKS1 (JBTS28/BBS13/MKS1)	ARL13B (JBTS8) OFD1 (JBTS10) TTC21B (JBTS11/NPHP12/SRTD4) KIF7 (JBTS12) TCTN1 (JBTS13) TMEM237 (JBTS14) CEP41 (JBTS15) TMEM138 (JBTS16) TCTN3 (JBTS18/OFD4) ZNF423 (JBTS19/NPHP14) TMEM231 (JBTS20/MKS11/OFD3) PDE6D (JBTS22) CEP104 (JBTS25) KIAA0556/KATNIP (JBTS26) B9D1 (JBTS27/MKS9) TMEM107 (JBTS29/MKS13) ARMC9 (JBTS30) CEP120 (JBTS31/SRTD13) SUFU (JBTS32) PIBF1 (JBTS33) B9D2 (JBTS34/MKS10)
Bardet–Biedl syndrome(70–80% rate of genetic proof)	**Most common genes**	**Less common genes**
	BBS1 (20–30%) BBS2 (15%) BBS7 (15%) BBS4 (8%) PTHB1 (BBS9) (8%) BBS10 (20–30%)	ARL6 (BBS3) BBS5 MKKS (BBS6) TTC8 (BBS8) TRIM32 (BBS11) BBS12 (BBS12) MKS1 (BBS13/JBTS28/MKS1) CEP290 (BBS14/NPHP6/SLS6/JBTS5/MKS4) WDPCP (BBS15) SDCCAG8 (BBS16/NPHP10/SLS7) LZTFL1 (BBS17) BBIP1 (BBS18) IFT27 (BBS19) IFT74 (BBS20) C8orf37 (BBS21)
Meckel–Gruber Syndrome(60% rate of genetic proof)	**Most common genes**	**Less common genes**
	MKS1 (MKS1/JBTS28/BBS13) (~7%) TMEM67 (~16–50%) (MKS3/NPHP11/JBTS6/COACH) CEP290 (MKS4/NPHP6/SLS6/JBTS5/BBS14) RPGRIP1L (MKS5/JBTS7/NPHP8/COACH)	TMEM216 (MKS2/JBTS2) CC2D2A (MKS6/JBTS9/COACH) NPHP3 (MKS7/NPHP3) TCTN2 (MKS8/JBTS24) B9D1 (MKS9/JBTS27) B9D2 (MKS10/JBTS34) TMEM231 (MKS11/JBTS20/OFD3) KIF14 (MKS12) TMEM107 (MKS13/JBTS29)
Short rib thoracic dysplasia with or without polydactyly	IFT80 (SRTD2) DYNC2H1 (SRTD3) TTC21B/IFT139 (SRTD4/JBTS11/NPHP12) WDR19/IFT144 (SRTD5/NPHP13/SLS8) NEK1 (SRTD6) WDR35 (SRTD7) WDR60 (SRTD8) IFT140 (SRTD9) IFT172 (SRTD10/NPHP17) WDR34 (SRTD11) CEP120 (SRTD13/JBTS31) KIAA0586 (SRTD14/JBTS23)	

## Genetic and Phenotypic Heterogeneity

**Figure 1:**
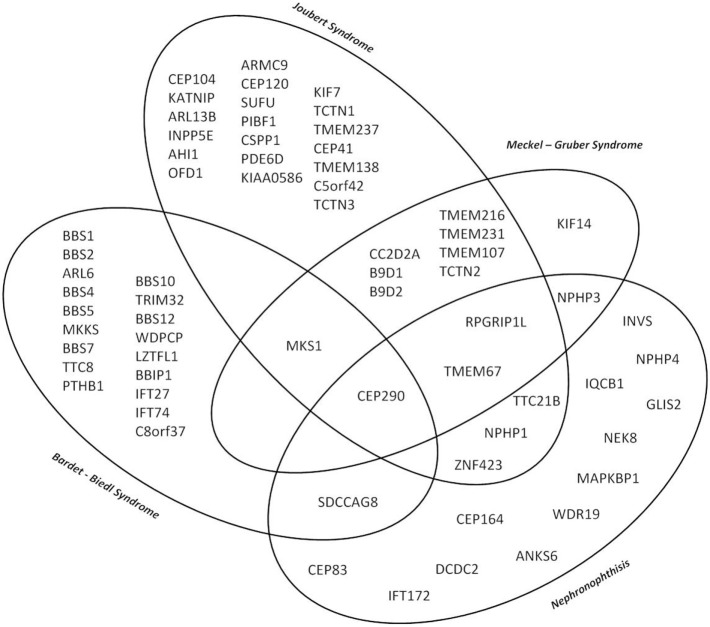
Venn diagram illustrating the major genetic and phenotypic overlap in hereditary ciliopathies featuring cystic kidney disease. So far well-defined clinical entities can be caused by mutations in multiple genes, whereas at the same time mutations in the same gene can cause very different phenotypes depending on the type of mutation ranging from isolated nephronophthisis (NPH) to lethal early embryonic multivisceral manifestations like Meckel–Gruber syndrome.

Although the clinical characteristics of the different hereditary cystic kidney diseases appear quite discriminative, there is significantly genetic as well as phenotypic overlap that hampers an early diagnosis and an individual clinical management (Figure 1) (1). The tremendous progress that has been achieved within the genetic field had major impact on the classification of cystic kidney diseases. However, the newly generated insights seem to further complicate the clinical situation for physicians dealing with affected individuals: it has become increasingly evident that so far well-defined clinical entities can be caused by mutations in multiple genes. Even in ARPKD, which for a long time has been assumed to be a single gene disease, modern NGS-based sequencing techniques just recently were able to identify a new genetic cause encoding a ciliary transition zone protein (26). Also, mutations in the same gene can cause very different phenotypes that range from lethal early embryonic multivisceral manifestations to single organ involvement starting in adolescence (Figure 1) (1, 27). Thus, recent advances in genomics challenged the classical Mendelian conditions and highlighted the genetic complexity of hereditary cystic kidney diseases and related ciliopathies (28). This complexity has been attributed to allelic heterogeneity, locus heterogeneity, reduced penetrance, variable expressivity, modifier genes, and/or environmental factors (29).

Based on these newly generated insights it has become clear that the “diagnostic odyssey” experienced by patients does not end with the identification of a disease-causing genotype (30). Neither does the traditional approach using textbook signs nor symptoms to guide diagnosis and management seem to be sufficient any longer (31). Rather a systematic and deep phenotyping approach supplementary to careful genotyping is critical in order to discover nonobvious phenotypes and to determine a precise diagnosis. Thus, physicians addressing hereditary cystic kidney diseases should combine both—detailed, multisystemic phenotyping and careful assessment of genotype pathogenicity—in order to capture all facets of the underlying disease (30). Against this background, it will be important to overcome the financial restraints associated with modern NGS-based sequencing currently hampering a comprehensive genetic characterization.

## Molecular Understanding

**Figure 2:**
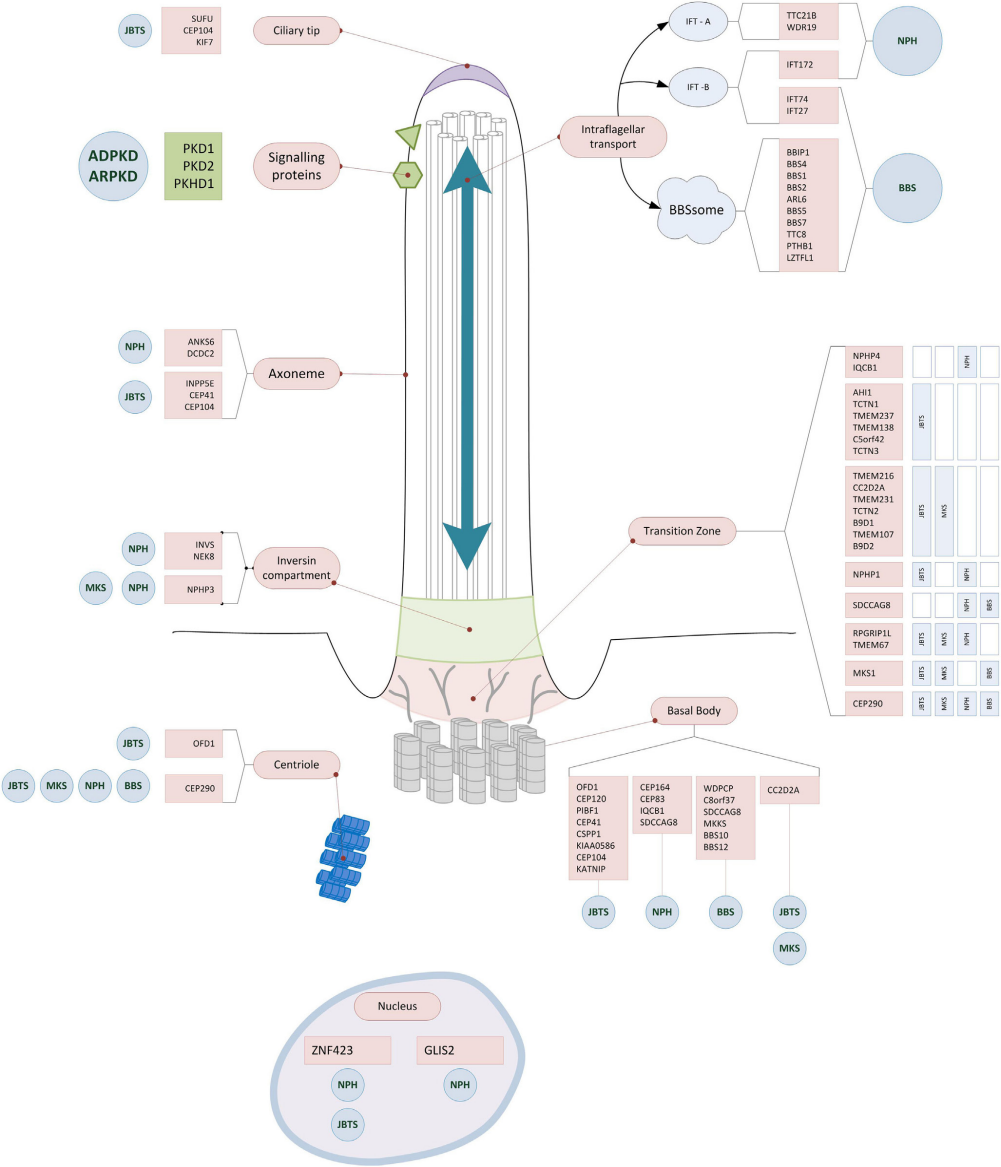
Ciliary localization of the proteins encoded by genes causing early onset cystic kidney diseases. Almost all gene products mutated in hereditary cystic kidney diseases are located at the primary cilium, the ciliary base, or the basal body. Some of the proteins form functional units (Polycystin1/2; BBSom; Nephrocystin-4,5) and are found at characteristic parts of the cilium (e.g., ciliary base). While for some of the gene mutations there is a clear impact on regular ciliary function (e.g., disrupting intraflagellar transport), for most others the exact pathophysiological mechanisms still remain to be unraveled. Abbreviations: NPH, nephronophthisis; JBTS, Joubert syndrome; BBS, Bardet–Biedl syndrome; MKS, Meckel–Gruber syndrome; ARPKD, autosomal recessive polycystic kidney disease; ADPKD, autosomal dominant polycystic kidney disease.

The genetic discoveries revolutionized our molecular understanding of cystic kidney diseases. The observation that most genes that have been identified so far encode proteins that co-localize in primary cilia and even interact as functional ciliary clusters suggested the existence of a common pathophysiological pathway and lead to the so-called ciliary hypothesis (Figure 2) (32, 33). However, different molecular mechanisms have been described to be altered in renal cyst formation, including cell-proliferation, apoptosis, DNA repair, fluid secretion into the cyst lumen, altered apico-basal cell polarity, directional cell migration, cell–cell adhesion, interaction with the extracellular matrix and ciliary function. Additionally, various intracellular signaling pathways were identified to be activated in epithelia developing cystic lesion, such as intracellular calcium signaling, cAMP triggered fluid secretion, the wnt-, hedgehog, mTOR-, notch-, YAP-hippo-, and other pathways. Whether or not these observations are related to each other and how the mentioned mechanisms could be connected with an altered ciliary function remains poorly understood and is the topic of ongoing research. Details on these topics are beyond the scope of this perspective. However, this has been excellently reviewed by Ong et al. recently (34–36).

## Network for Early Onset Cystic Kidney Diseases (NEOCYST)—A Comprehensive Approach on Hereditary Cystic Kidney Diseases

Despite all the achievements mentioned above, enormous challenges remain to be solved: the complex phenotypic spectrum, genetic heterogeneity, a lack of reliable genotype–phenotype correlations, a limited molecular understanding and the absence of disease-specific biomarkers still hamper an early diagnosis and individual counseling. Data from international registry studies like the NEPHREG or the ARegPKD registry will certainly help to define robust genotype–phenotype associations by increasing sample sizes and following individual disease courses in a longitudinal fashion (10, 37). However, in order to achieve a deeper clinical as well as molecular understanding, international collaborative efforts comprising various subspecialists will be necessary to move knowledge forward especially in the context of rare diseases. A detailed molecular understanding will be fundamental in order to identify new therapeutic targets and develop disease specific treatment approaches. Therefore, future research activities should go one step further and try to verify the hypotheses gained from cell culture experiments in specimen harvested from actual patients.

To address all these questions, the NEOCYST was initiated in 2016 in order to match the ongoing “revolution” in genetics and molecular biology with an improved efficacy of clinical data collection. Here, we would like to introduce the structure, the goals, and the individual work-packages of the NEOCYST collaborative accompanied by a short overview on the current state of progress.

## Design

Network for early onset cystic kidney diseases is a multidisciplinary, multicenter observational study of hereditary cystic kidney diseases in childhood. It combines a detailed and comprehensive clinical data collection with translational scientific approaches covering the genetic, molecular, and functional background.

## Aims

The primary goal of the NEOCYST consortium is to improve the clinical situation and the management of children with hereditary cystic kidney diseases. In order to reach this goal a multimodal approach was chosen including:

- A detailed and comprehensive longitudinal characterization of different hereditary cystic kidney diseases in childhood.
- The establishment of reliable genotype–phenotype correlations.
- The identification of new cystic disease genes.
- An improved molecular understanding.
- The identification of disease-specific marker proteins.
- A standardized clinical management based on standard-of-care-guidelines.
- The implementation of a national biobank generating a platform for future research projects.

## Study Cohort

Eligible are patients with the diagnosis of ARPKD, isolated NPH or NPH-related ciliopathies, BBS, and HNF1B-nephropathy. While for the first four study, inclusion is justified either by clinical or genetic criteria, HNF1B-nephropathy has to be confirmed genetically in order to avoid phenotypic overlap with other urinary tract malformations. For the clinical diagnosis of NPH at least two of the following criteria have to be met: (i) characteristic clinical course with polyuria/polydipsia; (ii) chronic kidney disease; (iii) kidney ultrasound or biopsy suggestive of NPH (24); (iv) pedigree compatible with autosomal recessive inheritance. The clinical diagnosis of BBS is based on the diagnostic criteria according to Beales et al. (38). Phenotypes reassembling a NPH-related ciliopathy are classified by the clinical criteria published by Bergmann (1).

Exclusion criteria encompass the definite genetic or clinical diagnosis of other cystic kidney diseases, in particular autosomal dominant polycystic kidney disease (ADPKD) which is addressed by other clinical registry studies.

Patient inclusion is preceded by a written informed consent and an approval by the local ethics committee of each contributing center, guaranteeing accordance with the principles of the Declaration of Helsinki and Good Clinical Practice guidelines.

## Work Packages

**Figure 3:**
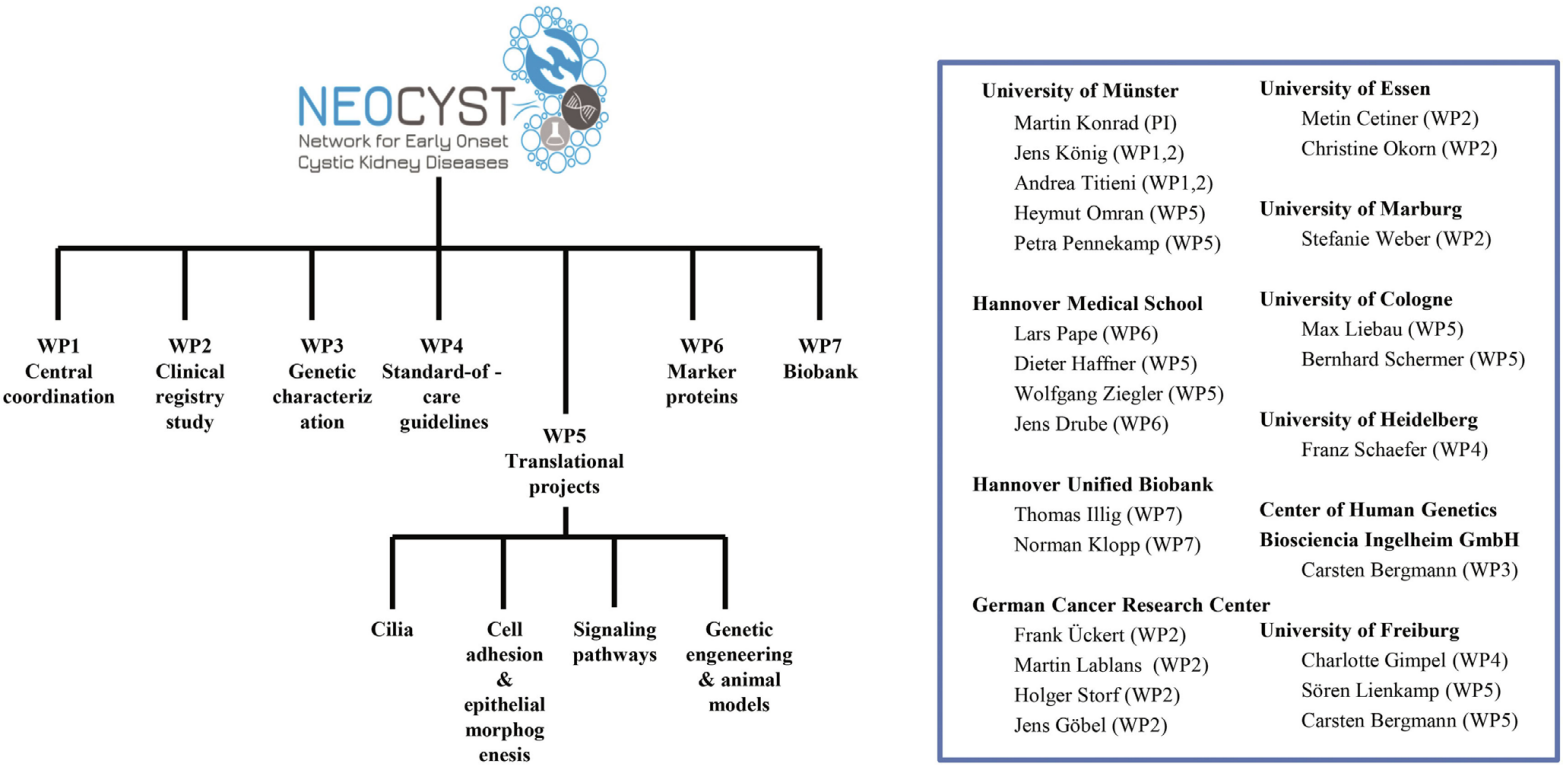
Organizational structure of the network for early onset cystic kidney diseases (NEOCYST) collaborative.

To address the goals mentioned above the NEOCYST consortium comprises seven work packages (Figure 3). Subsequently, we will outline the main topics covered by these work-packages:

- Clinical registry study: the web-based NEOCYST registry is a newly created retro- and prospective clinical registry providing a detailed genetic and phenotypic characterization of all hereditary cystic kidney diseases in one common database collecting cross-sectional as well as longitudinal data. Three pre-existing registries on NPH-RC,^1^ BBS, and HNF1B were merged to build the fundaments of this new database. Additionally, technical bridgehead components have been implemented that allow a direct comparative data analysis with the international ARPKD registry^2^ (37). Thereby, NEOCYST is the first registry study providing a comprehensive approach to early onset hereditary cystic kidney diseases and allowing back-to-back analyses on similarities and differences of the individual disease entities. At the same time, the database supplies technical features that enable external international cooperation and provide a platform for further research projects and future clinical trials throughout Europe.
- Genetic characterization: as the majority of NPH/NPH-RC patients as well as some ARPKD and BBS patients are still genetically unsolved, the NEOCYST network has set out to genetically characterize as many patients as possible, including potential genetic modifiers. The genetic approach applied consists of NGS-based panel diagnostics covering more than 100 ciliary genes followed by whole exome sequencing for those patients in whom no convincing mutations can be identified.
- Molecular biology: the molecular focus of the NEOCYST collaborative is on the characterization of an altered ciliary structure and function, intracellular signaling pathways as well as cell programming and cell adhesion processes. These topics get addressed by internationally acknowledged experts in the field of hereditary cystic kidney diseases (for details see NEOCYST consortium section below). Using conventional cell cultures as well as urine-derived renal epithelial cells (UREC) from patients carrying different mutations, modern techniques, such as proteomics, 3D spheroid models, cell (re)programming, and genomic engineering are applied to analyze different signaling pathways and their impact on cystogenesis *in vivo* and *in vitro*. Additionally, in order to characterize altered ciliary processes in hereditary cystic kidney diseases, nasal brushes from affected patients are generated and techniques like high-frequency video microscopy, freeze-fracture analysis, and immunohistological staining are applied to motile as well as immotile cilia. Finally, it is a goal to identify disease-specific proteome marker profiles from patients’ spot urine using a blinded prospective approach based on capillary electrophoresis coupled with mass spectrometry.
- Biobank: due to the paucity of patients characterized by an early onset renal failure, biological samples of such patients are highly precious. Thus, to ensure current as well as future research projects, the NEOCYST collaborative includes a biobank for storing urine, blood, and respiratory epithelial cells. All samples get stored at the Hannover Unified Biobank, one of the most modern biobanks in Europe. It is characterized by a high degree of automation and state-of-the-art biobank infrastructure according to standard operating procedures with data safety concepts, pseudonymization tools, sample identification *via* 2D bar codes and integrated IT systems that enable high quality and safety standards.
- Standard-of-care guidelines: so far, the clinical management of patients with hereditary cystic kidney diseases is mainly based on local physician’s experience and differs quite considerably between different centers. Due to the limited number of patients, the experience of most clinical centers is based on only a few single cases and clinical guidelines on the management of hereditary cystic kidney diseases are scarcely available. Thus, NEOCYST has set itself the task to develop and elaborate recommendations on different topics concerning hereditary cystic kidney diseases based on the given evidence in the literature as well as experts’ opinions.

## Current Status

The NEOCYST project started in February 2016 after initiation and approval of funding by the Federal Ministry of Education and Research (BMBF). After 24 months crucial milestones have been reached.

First of all, a homepage^3^ has been installed addressing patients, clinicians, and researchers to the same extent. Valuable information on the NEOCYST collaborative, the study goals as well as the medical background is provided in German and English language. A download domain contains all necessary study documents.

Furthermore, in September 2017 the technical implementation of the new online NEOCYST database has been completed merging three pre-existing clinical registries. Up-to-date 344 patients have been included comprising 194 patients with NPH and related ciliopathies, 88 patients with BBS, and 62 patients with HNF1B nephropathy. Especially, regarding HNF1B a rapid increase of participants has been observed ever since HNF1B became a part of the regular genetic screening in patients with hereditary cystic kidney diseases. However, we still assume a high number of unreported cases anticipating a further expansion of the study cohort within the next few years. The database is accessible *via* the NEOCYST-homepage (see text footnote 3).

Major success has been achieved by the genetic workgroup in identifying *DZIP1L* as a new cause for early onset cystic kidney diseases. *DZIP1L* encodes a protein located at the ciliary-transition-zone leading to a phenotype mimicking ARPKD when mutated (26). Additionally, mutations in *MKS1* so far only associated with lethal Meckel–Gruber syndrome were found also to be responsible for a milder phenotype resembling Joubert syndrome accompanied by agenesis of the corpus callosum (39).

The number of biological samples stored in the biobank is continuously growing and substantial progress has been achieved by the projects attended to the molecular biology of cystic kidney diseases. Methods that have been established in another context were successfully transferred to the scientific issues addressed by the NEOCYST project. Detailed imaging and structural analyses of primary and motile cilia have been elaborated and brought to a new scale. First results suggest that structural abnormalities and differences in ciliary protein composition specific for the underlying genetic defect. Furthermore, urine-derived renal epithelial cells, URECs have been established as a valid model on studying cell polarity and epithelial morphogenesis in cystic kidney diseases producing distinguishable patterns of cell clusters. Most results are still preliminary and part of an ongoing research process that will be the subject of future publications.

So far, 19 manuscripts from the NEOCYST cohort have been published or accepted for publication—including a positional paper on the “perinatal management of early onset cystic kidney diseases” (40). Two further positional papers on “imaging of early onset cystic kidney diseases” and “management of early onset ADPKD” have been finalized and are about to be published.

## Conclusion

Since initiation in February 2016, the NEOCYST collaborative has made substantial progress in addressing clinical, genetic, and molecular questions related to hereditary cystic kidney diseases. However, the mentioned achievements just represent the first steps of an ongoing process and further scientific initiatives and funding as well as international cooperation will be needed in order to answer these questions. Thus, any participation by international centers is warmly welcome. By setting up the infrastructure of an international clinical registry accompanied by several biological projects and the longtime storage of biomaterial, NEOCYST provides a platform that guarantees a detailed collection of precious longitudinal clinical data going along with further high-quality research approaches and enabling future clinical trials.

## Neocyst Consortium

**C. Bergmann**, Ingelheim, Germany; **M. Cetiner**, Essen, Germany; **J. Drube**, Hannover, Germany; **C. Gimpel**, Freiburg, Germany; **J. Göbel**, Frankfurt, Germany; **D. Haffner**, Hannover, Germany; **T. Illig**, Hannover, Germany; **N. Klopp**, Hannover, Germany; **J. König**, Münster, Germany; **M. Konrad**, Münster, Germany; **M. Lablans**, Heidelberg, Germany; **M. C. Liebau**, Cologne, Germany; **S. Lienkamp**, Freiburg, Germany; **C. Okorn**, Essen, Germany; **H. Omran**, Münster, Germany; **L. Pape**, Hannover, Germany; **P. Pennekamp**, Münster, Germany; **F. Schaefer**, Heidelberg, Germany; **B. Schermer**, Cologne, Germany; **H. Storf**, Frankfurt, Germany; **A. Titieni**, Münster, Germany; **F. Ückert**, Heidelberg, Germany; **S. Weber**, Marburg, Germany; **W. Ziegler**, Hannover, Germany.

## Author Contributions

JK, AT, and MK drafted the manuscript. MK is the principle investigator and JK the central coordinator of the described consortium.

## Conflict of Interest Statement

The authors declare that the research was conducted in the absence of any commercial or financial relationships that could be construed as a potential conflict of interest.

## Footnotes

^1^www.nephreg.de.

^2^www.aregpkd.org.

^3^www.neocyst.de.
